# Clinical use of hyaluronic acid in andrology: A review

**DOI:** 10.1111/andr.13083

**Published:** 2021-08-02

**Authors:** Alessandro Zucchi, Fabrizio Ildefonso Scroppo, Paolo Capogrosso, Andrea Salonia, Jacopo Duante, Vittorio Bini, Giovanni Liguori, Riccardo Bartoletti

**Affiliations:** ^1^ Department of Translational Research and New Technologies in Medicine and Surgery University of Pisa Pisa Italy; ^2^ Department of Urology and Andrology Ospedale di Circolo and Macchi Foundation Varese Italy; ^3^ Urology dept. University Vita‐Salute San Raffaele Milan Italy; ^4^ Department of Medicine and Surgery Santa Maria della Misericordia Hospital University of Perugia Pisa Italy; ^5^ Department of Urology Cattinara Hospital University of Trieste Trieste Italy

**Keywords:** hyaluronic acid, penile enlargement, Peyronie's disease, premature ejaculation

## Abstract

**Background:**

Hyaluronic acid is a glycosaminoglycan widely used in the fields of orthopedics, ophthalmology, and aesthetic medicine due to its significant ability to reduce the synthesis of pro‐inflammatory proteins and its activity against oxidative stress, a feature of many degenerative illnesses.

**Objectives:**

The objective of the present review is to provide a comprehensive narrative review of the most recent literature on the use of hyaluronic acid in andrology in order to facilitate the use of this therapeutic device in the common clinical practice of many physicians. Specific conditions covered in the review are Peyronie's disease, premature ejaculation, and penile enlargement.

**Materials and methods:**

A broad and comprehensive literature search included Medline, EMBASE, and the Cochrane Libraries, with no time restriction up to December 2020 and restricted to English language publications. Unpublished studies were not included. The study was registered as “The role of hyaluronic acid in andrology: A systematic review and meta‐analysis” in PROSPERO with the ID CRD42021223416.

**Discussion and conclusion:**

Hyaluronic acid is a valid choice for the treatment of Peyronie's disease in terms of the resolution of the acute phase of the disease and of contributing to stabilizing the disease as a bridge to potential surgery. Data, furthermore, suggest that hyaluronic acid is frequently associated with an overall clinical improvement, allowing the patient to resume normal sexual activity.

With regard to premature ejaculation, data suggests hyaluronic acid‐based treatments were effective in prolonging intra‐vaginal ejaculation time. Furthermore, hyaluronic acid was found to be safe and well‐tolerated, with main adverse events limited to local discomfort, ecchymosis, papule formation, and glans numbness, all of which were reported to resolve spontaneously.

Last, with regard to penile enlargement, the overall perception of experts is that hyaluronic acid may be an extremely well‐tolerated compound with potential for application in specific areas of male sexual health that are often neglected as compared to more common, and relatively simpler to treat, conditions.

## 1 INTRODUCTION

Hyaluronic acid (HA) is a glycosaminoglycan and a major component of the extra‐cellular matrix of the mammalian. The wide use of HA in the fields of orthopedics, ophthalmology, aesthetic medicine, and more is mainly associated with its biological properties, including its significant anti‐inflammatory and anti‐oxidative stress properties, making it the ideal compound for the treatment of osteoarticular degenerative pathologies.^1^ Indeed, free oxygen radicals (ROS), such as hydrogen peroxide (H_2_O_2_), hypochlorite ion (ClO^–^), hydroxyl radical (UOH), and superoxide anion (O2 –), are involved in both intracellular signal transduction and cellular degenerative processes.^2^, ^3^ Several studies have shown that chondrocyte apoptosis and cartilage degradation be caused by high ROS production levels, hence contributing to the pathogenesis of osteoarthritis.^2^, ^3^, ^4^, ^5^, ^6^, ^7^ Numerous data show that HA reduces ROS levels at the synovial sites in cases of osteoarthritis, counteracting apoptosis and promoting cell survival.^1^, ^8^

In a physiological condition, the hyaluronate molecule is highly polar and water‐soluble. In the connective tissue, HA maintains hydration, turgidity, plasticity, and viscosity, due to its specific steric conformation, which allows the storage of a considerable number of water molecules. Furthermore, HA possesses favorable biomechanical characteristics, thus acting like an anti‐shock molecule and efficient lubricant (e.g., in the synovial fluid), preventing damage to cells by physical stress.^2^, ^3^ These characteristics, together with its hypoallergenic properties, make HA the ideal candidate for increasing soft tissue volume and to be used as a filler. Once injected, HA is gradually metabolized and then reabsorbed over varying time frames depending on the treated surface and the type of HA used.

Injective HA treatment in andrology is now widespread, but data is limited due to the lack of prospective randomized and controlled studies with large series and consistent follow‐up assessments.

In this paper, we performed a comprehensive narrative review of the most recent literature on the use of HA in andrology.

## 2 MATERIALS AND METHODS

A broad and comprehensive literature review included Medline, EMBASE, and the Cochrane Libraries, with no time period restriction up to December 2020 and restricted to English language publications. Keywords used were the following: “HA and andrology, HA and Peyronie's disease (PD), HA and premature ejaculation (PE), and HA and penile enlargement.” Unpublished studies were not included.

We first registered this study as “The role of HA in andrology: a systematic review and meta‐analysis” in PROSPERO with the ID CRD42021223416. However, at the time of the systematic review, the inclusion criteria set out in the PROSPERO protocol were not applicable due to the lack of randomized clinical trials. Furthermore, many clinical trials did not include a control group and did not present common outcomes which could be analyzed with comparable parameters. It was therefore impossible to proceed with a meta‐analysis model, and the panel opted for a narrative review method.

Three research teams were engaged in the literature search, each of which was constituted by two individuals each with specific clinical expertise. The groups independently focused on: i) PD, ii) PE, and iii) penile enlargement. After preliminary evaluation, if the research team was not in agreement on the article content, the article was re‐evaluated by an external reviewer (belonging to another of the three teams). The selected articles were then assessed in their full test and evaluated via the Risk Bias systems (Rob 2 tool). This further evaluation was then omitted due to difficulties in carrying out a true systematic review process. Tables 1, 2, 3 contain the selected papers in the three fields of interest.

**TABLE 1:** Hyaluronic acid treatment results in Peyronie's disease

Author	Groups	No. of patients	Type of study	Therapeutic scheme	Duration of treatment (weeks)	Follow up (months)	Plaque dimension (mm)	Recurvatum (°)	IIEF score	VAS score	PGI‐I score	Main outcome
Cai^29^	2	41 + 40	Randomized clinical trial	HA intralesional + oral	6	3	−3.0 ± 10	−7.8 ± 3.9	4 ± 0.3	−4.0 ± 2	1 ± 1	Modification in penile curvature
Cai^29^	“	“	“ “	HA intralesional	6	3	−2.0 ± 9	−4.1 ± 2.7	2 ± 0.5	−4.0 ± 2	3 ± 2	“ “
Cocci^28^	2	125 + 119	Prospective case‐control	HA intralesional (versus Verapamil)	8	3	−1.50 (r. 1.60–2.10)	–9.50 (r. 4.50–13.00)	1.0 (1.12–1.94)	−4.0 (4.11–3.65)		Penile curvature
Favilla^27^	2	69 + 63	Prospective case‐control	Ha intralesional (versus Verapamil)	12	3	−1.80 ± 2.47	–4.60 ± 5.63	1.78 ± 2.48		3.13 ± 1.53	Penile curvature
Gennaro^26^	2	83 + 81	Case‐control	Ha intralesional (versus no treatment)	26	6‐12‐24		−9.01 ± 7.58	+3.814 ± 3.013			Plaque size
Zucchi^25^	1	65	Prospective single arm	Ha intralesional (no control group)	10	2	−2 (r.1‐30)	−10 (r. 0–40)	+1 (15–25)	−2		Plaque size

**TABLE 2:** Hyaluronic acid treatment results in premature ejaculation (PE)

Author	Group	No. of patients	Type of Study	Terapeutic scheme	Self‐rated satisfaction (pre)	Self‐rated satisfaction (post)	IELT pre	IELT post	VT (mA) pre	VT (mA) post	Main Outcome
Littara^38^	1	110	Prospective single arm	1 ml HA	1.2 ± 0.04	5.3 ± 0.07	88.34 ± 3.14	293.14 ± 8.16			Intravaginal ejaculation latency
Alahwany^40^	2 crossover	15 + 15	Randomized clinical trial	1 ml syringes of crosslinked HA (TeosyalPureSense Global Action 25 mg/ml versus saline injections	1.4 ± 0.5	2.6 ± 1.5	33.5 ± 14.8	73 (0–240)			Intravaginal ejaculation latency
Kim^36^	3	25 + 49 + 65	Prospective non randomized case control	2 cc of injectable HA gel Perlane and Restylane		75% satisfaction	96.5 ± 52.32	281.9 ± 93.2	4.54 (3–7)	9.10 (8–12)	Intravaginal ejaculation latency
Kwak^37^	1	38	Retrospective	hyaluronic acid (HA) gel (Perlane)		76% satisfaction	84.2 (45–170)	376.7 (270–470)	3.44 (2–6)	9.72 (8–11 )	Intravaginal ejaculation latency
Abdallah^39^	2	30 + 30	Randomized clinical trial	2 ml of hyaluronic acid gel (Hyalift 3.5% micronised hyaluronic acid) versus 2 ml of hyaluronic acid gel using the multiple puncture techniques.		2.12 ± 1.16	7.71 ± 7.86				Intravaginal ejaculation latency

**TABLE 3:** Penile augmentation (PA) selected papers

Author	No. of patients	Main outcome	Type of study
Yang et al.^52^, ^53^, ^54^	39 + 35	Psychological effect of PA	Randomized clinical trial
Moon et al.^47^			Animal model
Ahn et al.^55^		Complications of PA	Narrative review
Kim et al.^48^	187	Maximum glans circumference	Randomized clinical trial
Micheels et al.^50^	12	Psychological effect of PA	Clinical trial
Kwak et al.^49^	41	Penile girth	Clinical trial
Moon et al.^44^			Review
Sito et al.^51^	56 + 27	Circumferential enlargement	Retrospective study

## 3 RESULTS AND DISCUSSION

### 3.1 Peyronie's disease

The etiology of PD is still largely unknown. Likewise, its complex pathogenetic mechanisms are not yet completely understood. However, in accordance with the different theories proposed by some authors, a single traumatic event or multiple and repeated micro‐traumas during sexual activity could provoke a prolonged inflammatory reaction to the fibers of the tunica albuginea, thus causing an autoimmune response.^9^, ^10^ This complex reaction includes several mechanisms such as micro‐vascular damage, accumulation of inflammatory cells and fibrin, over‐expression of cytokines, and growth factors that stimulate matrix protein production, mainly collagen, which is partially responsible for the formation of the plaques.^11^, ^12^, ^13^, ^14^, ^15^, ^16^ Disease progression may eventually lead to calcification or ossification of the plaque in 15%–25% of patients.^17^, ^18^

The commonly used pharmacological treatments depend on the stage of the disease, with the aim of reversing, interrupting, or attenuating the course of the disease, reducing deformity, and improving sexual function.^19^, ^20^ The latest updated AUA and EAU guidelines^21^, ^22^ confirm that no drugs have been approved for the treatment of Peyronie's disease, except for potassium para‐aminobenzoate (Potaba), considered “probably effective” by the FDA, and collagenase clostridium histolyticum, which is no longer available in the European market. Therefore, new drugs capable of solving painful symptoms, blocking fibrosis progression, reducing plaque size and degree of recurvatum, with the aim of restoring satisfaction during sexual intercourse, are needed.

Commonalities can be found between the cellular and molecular players involved in the complex pathophysiology of PD and in inflammatory/degenerative osteoarticular pathologies. Indeed, the same mediators cause an inflammatory milieu and oxidative stress on the tunica albuginea, eventually causing a degenerative process that is difficult to reverse. Indeed, during the initial stages of this disease (i.e, acute phase) the increased levels of oxidative stress induce the overexpression of fibrogenic cytokines along with an increased synthesis of collagen.^23^, ^24^

In this context, the use of HA has rapidly become widespread in recent years because of its anti‐inflammatory and viscoelastic properties (Table 1).^25^, ^26^ Indeed, Zucchi et al. reported data of 65 patients with PD treated with HA injections (0.8% highly purified sodium salt HA 16 mg/2 ml; IBSA Farmaceutici Italia, Lodi, Italy) in a single‐arm, self‐controlled, and interventional study.^25^ The study showed encouraging results in terms of reduction in the size of the fibrotic plaque, penile recurvatum, and an improvement in terms of patients' overall sexual satisfaction. The study was mainly limited by the lack of a control group and the short follow‐up (namely 3 months).^25^ Similarly, Gennaro et al.^26^ reported the experience conducted on 83 PD patients treated for 6 months with 20 mg HA (Hyalgan Fidia) penile injections (30 injections, 20 mg/2 ml in total over a 6‐month period) with 24‐month follow‐up. Compared to a non‐treated control group, at the 12‐month assessment, there was an improvement (reduction) in the size of the plaque, the severity of the recurvatum as well as in overall sexual performance. After 24 months there was no further improvement, thus demonstrating that the disease had remained stable.

Favilla et al.^27^ published the findings of a double‐blind, randomized, multicenter study with 63 patients treated with HA (0.8% highly purified sodium salt HA 16 mg/2 ml; IBSA Farmaceutici Italia) compared to intralesional Verapamil 10 mg. The primary endpoint was the change from baseline in the degree of penile recurvatum at 12 weeks after therapy. Secondary endpoints were the change in plaque size, International Index of Erectile Function‐5 score (IIEF), and Patient Global Impression of Improvement score (PGI‐I). The results showed that HA was superior to Verapamil in terms of plaque size reduction and degree of curvature, as well as sexual activity improvement and overall patient satisfaction. The study was limited by the absence of a placebo group, only 3 months of follow‐up, and the subjective variables associated with the plaque measurement in the 11 Italian centers enrolled.

Similar results were published by Cocci et al.^28^ in a prospective, non‐randomized study, comparing two groups of patients affected by PD and treated respectively with a course of HA infiltrations (0.8% highly purified sodium salt HA 16 mg/2 ml) and with Verapamil 10 mg. The IIEF, plaque size, degree of curvature calculated with a goniometer at maximum penile rigidity were used as outcome parameters. Furthermore, a subjective self‐assessment of penile pain was performed using VAS scales. Data showed a post versus baseline significant reduction in terms of plaque size, recurvatum, and IIEF‐15 improvement in patients treated with HA injections, along with significantly better results compared to the group treated with Verapamil. One major limitation was the lack of a control group of placebo‐treated patients.^28^ Finally, Cai et al^29^ published a prospective randomized phase III study with 81 PD patients enrolled at two centers and randomized to oral HA administration (a combination of extracts of HA, avocado, and soy unsaponifiable substances) combined with intralesional HA (0.8% highly purified sodium salt HA 16 mg/2 ml) (*n* = 41 patients) as compared with intralesional treatment only (*n* = 40 patients). Outcomes considered were changes in penile curvature, IIEF, and PGI‐I questionnaire. In addition, a VAS (ranging from 0 to 10) was applied to self‐assess penile pain. The study showed that the combination of oral and intralesional HA promoted better results in terms of curvature modifications and overall sexual satisfaction (*p* < 0.001).

**FIGURE 1:**
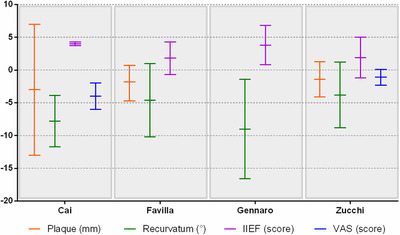
Different results using hyaluronic acid in the treatment of Peyronie's disease

Almost all the aforementioned studies had a prospective randomized design, and all showed a volumetric reduction of the plaque and the severity of the curvature (Figure 1). Likewise, the studies also outlined an improvement in terms of IIEF and sexual satisfaction. Although the series of patients presented are relatively large, the lack of formal randomization versus adequate control groups, as well as the restricted length of the follow‐up periods, are major methodological flaws of all studies.

In conclusion, HA is emerging as a valid choice for the treatment of PD in terms of resolution of the acute phase of the disease, and it is plausible to posit that the use of HA may contribute to the stabilization of the disease, an indispensable requirement for the subsequent choice of a possible surgical strategy. Even more clinically relevant, the observed findings suggested that HA was associated with an overall improvement of the clinical picture which, in most cases, allowed the patient to resume an almost normal sexual activity.

### 3.2 Premature ejaculation

PE is defined by the International Society of Sexual Medicines as an ejaculation that always or nearly always occurs before or within about 1 min of vaginal penetration from the first sexual experience (lifelong PE), or a clinically significant reduction in latency time, often to about 3 min or less (acquired PE).^30^ Moreover, PE is usually considered to be associated with emotional distress and bother.^31^ Population‐based studies have shown a prevalence of PE ranging from 8% to 30% of the male population.^32^ The pathophysiology of PE has been associated with an underlying neurobiological functional disturbance characterized by an alteration of the 5‐hydroxytryptamine neurotransmission leading to a lower ejaculatory threshold.^33^ Moreover, both psychological and organic factors including urinary tract infections and metabolic alteration could play a role.^33^ To date, numerous treatment modalities have been implemented for PE, including topical anesthetic agents, on‐demand, on label, and continuous off‐label selective serotonin reuptake inhibitors, and tramadol.^34^ Notwithstanding, all these treatments have shown to be variably effective, and discontinuation rates are still high.^35^, ^36^

The injection of HA as a bulking agent within the glans has been suggested as a potential local treatment for PE, with the goal to act as a barrier inhibiting the tactic stimuli to access the receptor, thus delaying the ejaculatory reflex.^36^ To date, three prospective single‐arm studies,^36^, ^37^, ^38^ one randomized non‐controlled trial,^39^ and one randomized controlled trial^40^ have investigated the efficacy and safety of HA glans injection in men with PE (Table 2). Kim et al.^36^ firstly reported data of 139 PE patients treated with either dorsal neurectomy (Group 1); dorsal neurectomy + HA glans augmentation with HA injections (Group 2); or glans augmentation with HA only (Group 3). For glans augmentation, 2 cc of HA was injected subcutaneously at one‐third from the tip of the glans toward the coronal sulcus using a “Fan” technique by rotating the needle to both sides during the injection. At 6‐month follow‐up, the authors showed a significant increase in post‐treatment intra‐vaginal ejaculatory times (IELT) compared to baseline in all groups (Group 1: 89.2 versus 235.6 s; Group 2: 101.5 versus 324.2 s; Group 3: 96.5 versus 281.9 s). The same authors reported results of a 5‐year follow‐up in 38 patients^37^. A decrease of IELT was depicted as compared with the 6‐month assessment, but it was still longer compared to baseline. Moreover, they reported that patients had a satisfactory sexual life (as assessed with a single question) in 76% of cases. A few years later, Abdallah et al. investigated the effect of HA glans injection in a pilot study conducted in 60 PE patients.^39^ They performed a randomized non‐controlled trial to assess the effect of two different techniques of HA injection: the previously described “fan” technique (Group A) and a multiple puncture technique (Group B), where the HA gel was administered via multiple injections within the glans. The latter technique was deemed to allow for a better distribution of the drug in the tissue. At 1‐month follow‐up, the IELT significantly increased in both groups (2.12 ± 1.16 to 7.71 ± 7.86 min), then slightly dropping after 3 months (5.32 ± 3.52 min); no significant difference was observed between the two groups. Similar results were reported by another single‐arm trial with data from 110 PE patients treated with multiple HA gel injections within the glans and showing an increase in IELT from 88.34 ± 3.14 to 293.14 ± 8.16 s, 6 months post‐treatment.^38^ Finally, Alahwany et al. performed a controlled cross‐over study randomizing 30 PE patients to receive either HA glans injection or saline injection.^40^ Results showed a significant IELT improvement after 1 month in the treatment group; the magnitude of increase was a median of 2.6 fold higher than baseline compared to 1.1 fold for patients receiving saline (*p* = 0.001). In detail, the increase in IELT was observed in 67% of patients, while 33% did not report any change after HA treatment. Moreover, median scores of the Arabic Index of Premature Ejaculation, assessing patients and partner distress, were also increased at 1‐month follow‐up in the treatment group.

**FIGURE 2:**
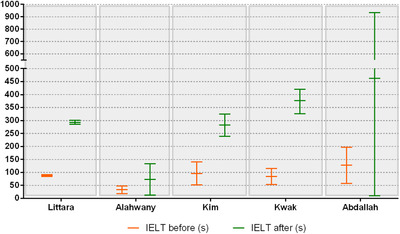
Different results using hyaluronic acid in the treatment of premature ejaculation

Overall, data suggested that HA treatment for PE was effective in improving IELT (Figure 2), safe and well‐tolerated, with reported side effects ranging from 0% to 30% across all studies. The main adverse events were local discomfort, ecchymosis, papule formation, and glans numbness, all resolving reported to resolve spontaneously.

### 3.3 Penile augmentation

The size of the penis has kept its subjective and popular importance over time, with adequate dimensions corresponding to the idea of advantages both from an aesthetic and a sexual standpoint.

A variety of surgical and physiotherapy techniques have been proposed throughout times to achieve lengthening and increasing the girth of the penis (i.e., penile girth enhancement) and the glans (i.e., glans penis augmentation). Such techniques have been characterized by wide limitations, mainly related to methodological biases of the proposed studies, frequent complications, not always satisfactory aesthetic outcomes, as well as the often unrealistic expectations of the patient.^41^, ^42^

In this context, while the use of various fillers has gained wide popularity with the aim of increasing the size of soft tissues in dermatology and aesthetic medicine,^43^ the application of fillers intended for penile girth enhancement and glans penis augmentation posed particular problems in consideration of the specific anatomy of the penile shaft and, to date, it has not yet been established which should be the ideal filler dedicated to the purpose (Table 3).

By virtue of its molecular characteristics, HA is a potential candidate to be considered in the cosmetic and reconstructive andrological area, not only in hard penis plastics but also for penile “cosmetic” techniques. In fact, HA is a ubiquitous component of connective tissue and in the intercellular matrix of the dermis, thus presenting a high biocompatibility profile that limits its antigenic properties and foreign body reactions.^44^

Historically, the first attempts of enlargement phalloplasty considered surgical techniques of lengthening and girth increasing using autologous dermofat grafts, with often unsatisfactory results due to the development of nodularity, deformity of the penile skin, infections, coupled with suboptimal long‐term outcomes because of the loss of almost 55%–90% of the initial volumetric increase after one year since surgery.^45^ In 1998, Sito and Sorrentino, in order to increase penile circumference, suggested the injection of bovine collagen into the coronal sulcus on the sheath of the penis, again with unsatisfactory aesthetic results.^46^ In 2002, based on animal models, Moon et al.^47^ reported the possibility of implanting HA gel subcutaneously in the glans. Thereafter, Kim et al.,^48^ in a large series of 187 patients injected 2 cm^3^ of HA gel (Perlane; Q‐med, Uppsala, Sweden) subcutaneously at the proximal one‐third from the tip of glans to coronal sulcus. A second injection of Restylane (HA gel; Q‐med, Uppsala, Sweden) was performed to correct potential anomalies of the glans surface after the first procedure. One year after, the net increase in maximum glandular circumference was 14.93 ± 0.80 mm in a group of naïve patients (Group 1, *n* = 100) compared to and of 14.78 ± 0.89 mm in 87 patients (Group 2) treated with a previous unsatisfactory dermofat graft. At 1‐year after the injection, more than 50% of the injected volume was maintained in 95% of Group 1 and 100% of Group 2, respectively, as for subjective patient's visual estimation. Moreover, the percentage of postoperative satisfaction assessed by VAS (Gr 0–4, min to max) was 77% in Group 1 and 69% in Group 2 (Gr 3, 4), with no serious treatment‐emergent adverse events. Kwak et al.^49^ examined the effects of high‐density HA (Restylane SubQ; Q‐med, Uppsala, Sweden) injected with lidocaine into the fascial layer of the penile shaft in 50 patients with “subjectively small” penis, with the aim of increase PGE. Forty‐one patients completed the follow‐up at 18 months. Compared to a basal circumference of 7.48 ± 0.35 cm, a significant increase in the maximum penile circumference was obtained up to 11.41 ± 0.34 cm at 1 month (*p* < 0.0001), with results substantially unchanged after 18 months (11.26 ± 0.33 cm). The findings were confirmed using a VAS of the residual volume of the penis at the latest follow‐up assessment, and with the degree of satisfaction reported both by the patient and the patient's partner. Micheels et al.^50^ essentially resumed the technique proposed by Sito defined as the “Mushroom technique”, with injections applied circumferentially around the corona of the glans (Fly Agaric) of high‐density HA gel (Belotero Basic) in 12 subjects and, in some cases, to the surface of the glans (Sex Toys effect) or alternatively by retrograde contiguous injections into the corona of the glans. One milliliter of gel was initially injected, then followed by a second injection of 1 ml after 1 to 2 months and by the third injection of a more‐volumizing HA (Belotero Intense) at 3 months, whenever requested by the patient. Study participants were offered a multiple‐choice self‐assessment questionnaire. All patients reported an improvement in local sensitivity and an increase in terms of glans diameter, with the persistence of raised papules on the crown of the glans for a period of at least 12 months. Side effects were bruises and some bleeding (one patient) and pain soon after the procedure (one patient53‐In a further retrospective study conducted on 83 patients, Sito et al.^51^ compared Macrolane (Q‐Med AB, Uppsala, Sweden), a slow resorption HA gel (12–18 months) infiltrated with an emicirconferential‐injection filling of the penis, with lipofilling. The increase in penile circumference obtained with both techniques varies from 3.2 to 4.5 cm in a flaccid state. While the authors did not observe complications in HA‐treated patients, eight lipofilling‐treated patients developed granuloma and, in one case, lipid necrosis. Yang et al.^52^ conducted the first randomized, multicenter, patient/evaluator‐blinded study comparing the effectiveness of the injection of HA gel fillers (Chaeum Shape; Across Co. Ltd., Chuncheon, Korea) and polylactic acid (PLA) for penile augmentation in 72 patients with “small penis syndrome”, with 48‐week follow‐up. A lasting increase was obtained in both groups (16.95 ± 10.53 and 13.49 ± 9.98 mm of mean increase in the HA and PLA groups, respectively; *p* < 0.001). Interestingly, after 4 weeks from treatment, results significantly improved after filling with HA than PLA (*p* < 0.001), while no significant differences were observed at 48 weeks. Similarly, in terms of the degree of satisfaction for penile appearance assessed by a VAS score, the authors reported an increase after the injection, which was maintained at the end of the follow‐up without differences in the two groups. The only adverse event in HA‐treated patients was injection site induration in one patient only. The same Author substantially confirmed these results in further studies.^53^, ^54^

As for the potential side effects related to the use of glans fillers, Ahn et al.^55^ underlined how the use of HA has gained popularity by virtue of a favorable safety profile, linked to the characteristics of biodegradability and reabsorption overtime of the compound. Conversely, non‐absorbable fillers that increase the longevity of HA can cause complications immediately after injection or even several years after implantation, such as necrosis secondary to inflammation and vascular compromise, granulomas, and nodule formations. The most common complications are hypercorrection, surface irregularities, visibility of the filler, Tyndall effect, and granuloma.

## 4 CONCLUSIONS

This narrative review reports a summary of the available literature regarding the use of HA in andrology. The use of different types of HA injected in various anatomical sites with different dosages, different techniques and follow‐up assessments for distinct conditions do not allow a reliable comparison of the results. On the one hand, most studies are not randomized and without control groups, with an overall reduced level of evidence. On the other, there is a lack of homogeneity in assessed parameters. The latter point is exemplified, for instance, by the measurement of the degree of penis curvature and plaque size in men with PD, which prevents meta‐analysis of the available data. Moreover, in many studies on PE, improvement *per se* is an index of secondary outcomes after following the evaluation of penile augmentation techniques, thus conditioning the real perception of HA effectiveness for PE. Furthermore, the usefulness of self‐assessment scales for patients with “small penis” is limited by differences in self‐expectations. Last, to date, there is no single gold‐standard technique for every possible application of HA in the field of andrology; this major issue certainly promotes the need for rigorous randomized clinical trials dealing with standardized techniques, sites of injections, and substances to be infiltrated. However, the overall feeling is that HA may represent an extremely well‐tolerated compound with scope for effective application in specific areas of male sexual health that are often neglected as compared with more common, and relatively simpler to treat, conditions.

## CONFLICT OF INTEREST

The authors declare that they have no conflict of interest.

## AUTHOR CONTRIBUTIONS

Conceptualization: Fabrizio Scroppo and Alessandro Zucchi; Data curation: Riccardo Bartoletti and Alessandro Zucchi; Formal analysis: Vittorio Bini and Paolo Capogrosso, and Giovanni Liguori; Investigation: Jacopo Durante and Giovanni Liguori; Methodology: Riccardo Bartoletti and Alessandro Zucchi; Supervision: Riccardo Bartoletti and Andrea Salonia; Writing–original draft: Alessandro Zucchi and Fabrizio Scroppo; Review and editing: Alessandro Zucchi and Fabrizio Scroppo.
